# Feasibility of the REDCap platform for Single Center and Collaborative Multicenter CMR Research

**DOI:** 10.1186/1532-429X-16-S1-P89

**Published:** 2014-01-16

**Authors:** Timothy C Wong, Gaby Captur, Uma Valeti, James Moon, Erik B Schelbert

**Affiliations:** 1 University of Pittsburgh, Pittsburgh, Pennsylvania, USA; 2 University College London, London, UK; 3 University of Minnesota, Minneapolis, Minnesota, USA

## Background

REDCap (Research Enterprise Data CAPture) software may provide a feasible platform for CMR Centers to: a) capture clinical throughput securely for research purposes, and 2) collaborate using a common platform for either distributed or centralized data storage. REDCap may facilitate CMR Centers' participation in the research enterprise, especially those with limited resources. REDCap may catalyze multicenter studies with "distributed data collection" where CMR sites can clone shared data dictionaries across sites for subsequent compilation into a singular master data file.

## Methods

Investigators without prior REDCap training created a REDCap database hosted by the University of Pittsburgh with software developed by Vanderbilt University. In an IRB approved protocol. A full time research nurse consented patients referred for clinical CMR scans and abstracted patients' clinical data into REDCap during CMR scanning. Data elements included: key summary findings from CMR (volumes, LGE, etc) and all prior cardiac imaging data including full (unstructured) reports pasted into text fields or uploaded pdf attachments; blood testing acquired during IV placement; demographics; comorbidity; and medications. Front end quality assurance measures included data formats, data ranges, and redundant identifiers. We maximized data security by configuring users' rights, limiting data exports to the only the principal investigator, and logging every time stamped manipulation to the database. Adverse event queries occurred biannually. Data were entered via a web browser and stored on encrypted servers behind firewalls. Data were exported to statistical software packages for analysis.

## Results

**Figure 1:**
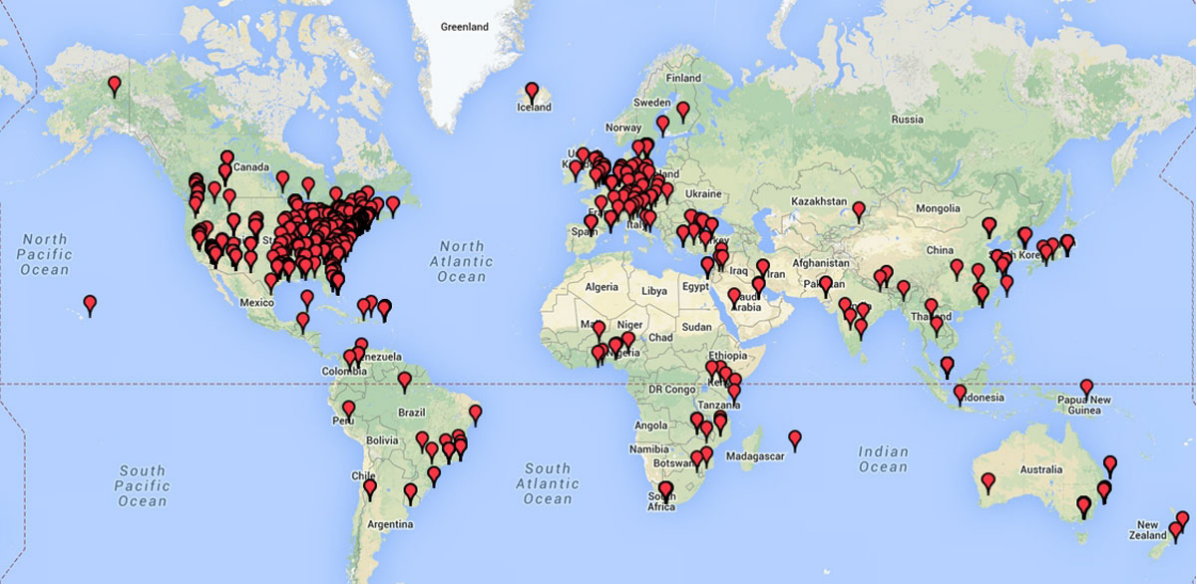
**REDCap platforms are available worldwide and provide pre-existing infrastructure thus simplifying multicenter collaborations**.

We established single center feasibility. We enrolled >3000 consecutive individuals over 3.5 years. This cohort formed the basis for several publications and ongoing investigations. No security breaches occurred. To demonstrate feasibility of multicenter data collection, we imported two entire REDCap project databases (field names and structure without data) from the University of Minnesota and the University College of London via email exchange of a .csv file. We cloned the databases rendering them operational in <5 minutes similar to a new collaborative project site. We also queried the world map of REDCap capable sites (http://www.project-redcap.org) to demonstrate the potential for global multicenter data collection (Figure 1).

## Conclusions

REDCap is widely available and provides a robust platform for clinical CMR research. REDCap provides sites with limited resources a powerful means for rich data collection for clinical CMR research. Scarce resources can then be directed to the burden of rich data collection necessary for robust risk adjustment and multiple hypothesis testing. Shared REDCap data dictionaries are feasible and have the potential to enhance collaboration. REDCap has the potential to accelerate clinical CMR research.

## Funding

American Heart Association The Pittsburgh Foundation.

